# Binding of cationic analogues of α-MSH to lipopolysaccharide and disruption of the cytoplasmic membranes caused bactericidal action against *Escherichia coli*

**DOI:** 10.1038/s41598-022-05684-z

**Published:** 2022-02-07

**Authors:** Kanchan Tiwari, Madhuri Singh, Prince Kumar, Kasturi Mukhopadhyay

**Affiliations:** Antimicrobial Research Laboratory, School of Environmental Sciences, Jawaharlal Nehru University, New Delhi, India

**Keywords:** Biophysics, Drug discovery, Microbiology

## Abstract

In earlier reports, we have shown the antimicrobial activity of a host neuropeptide, alpha-melanocyte stimulating hormone (α-MSH) and its cationic analogues against *Staphylococcus aureus*. These analogues of α-MSH showed enhanced staphylocidal activity without any significant mammalian cell toxicity. Therefore, here, we explored the antimicrobial activity of α-MSH and its cationic analogues against *Escherichia coli*. Though the presence of lipopolysaccharide (LPS) in Gram-negative bacteria enables them to resist most conventional antibiotics, encouragingly α-MSH and its four analogues showed killing of both logarithmic and stationary phase *E. coli* cells in a time, dose and cationicity-dependent manner. In fact, the most cationic analogue, KKK-MSH with a + 5 charge, demonstrated successful eradication of 10^5^ CFU/mL of *E. coli* cells within 15 min at a concentration as low as 1 µM. BC displacement experiment revealed that cationicity of the peptides was directly related to the killing efficacy of these α-MSH analogues against *E. coli* cells via initial LPS-binding, leading to rapid disruption of the LPS-outer membrane complex followed by inner bacterial membrane damage and eventual cell death. Here, we propose α-MSH based cationic peptides as promising future agents with broad-spectrum antibacterial efficacy against both Gram-negative and Gram-positive pathogens.

## Introduction

Despite the growing awareness, antimicrobial resistance (AMR) continues to increase and has become a global threat to public health, particularly after the upsurge of multidrug-resistant (MDR) strains^1^. The spread of MDR Gram-negative bacteria (GNB) is one of the most pressing emerging issues in recent years^2,3^, with nine of the twelve pathogens listed as public health threats by the World Health Organization being Gram-negative^4^. In general, GNB possess intrinsic resistance to many antibiotics because their outer membrane consists of lipopolysaccharide (LPS), which functions as a permeability barrier and prevents entry of many antibiotics into the cell^5^.

*Escherichia coli* (*E. coli*) is the most common GNB that usually present as harmless commensals of the gastrointestinal tract and forms part of the normal gut microbiota^6^. However, after prolonged exposure and under a stepwise selection of antibiotics, *E. coli* has developed resistance against various antibiotics, including aminoglycosides, fluoroquinolones, and β-lactams, and is now considered as one of the most difficult GNB pathogens to treat^7–9^. It is one of the most prevalent and leading causative agents of many common bacterial infections, including urinary tract infection (UTI), diarrhea, bacteremia, pneumonia, and meningitis^10,11^. Treating patients remains a serious challenge due to the limited arsenal of effective antibiotics with even the last line of active antibiotics, such as colistin and carbapenem, failing miserably against MDR *E. coli*, while the rate of the development of new antimicrobial agents is almost stagnant^12–14^. Thus, in this scenario, there is an urgent need for new antimicrobial agents that could treat resistant *E. coli* infections.

In the quest for an alternative to conventional antibiotics against MDR bacterial strains over the past decade, our lab has been exploring the antibacterial activity of a 13 amino acid long, human neuropeptide alpha-melanocyte stimulating hormone (α-MSH) against methicillin-resistant *Staphylococcus aureus* (MRSA)^15^, and has reported its antistaphylococcal activity to result from its multi-targeting mode of action, whereby after selectively penetrating the negatively charged bacterial membrane, it inhibited DNA replication and protein synthesis in *S. aureus* cells, causing cell lysis and death^16,17^. Moreover, structure–activity relationships showed that α-MSH(6–13)-based peptidomimetics with antibacterial and candidacidal activity were more potent than parent peptide^18,19^. Next, we developed several α-MSH analogues with enhanced cationic charge by replacing the neutral amino acids with Lysine (K) and replacing the anionic amino acids with alanine. These cationic α-MSH analogues exhibited improved staphylocidal activity through an increase in their interaction with anionic bacterial membranes without compromising their cell selectivity^20^. Thus, after establishing the antimicrobial efficacy of α-MSH and its modified analogues against the most challenging Gram-positive bacteria (GPB), i.e., MRSA, we became interested in exploring their efficacy against GNB pathogens. Here, we aimed to examine the antimicrobial activity and mechanism of action of α-MSH and its cationic analogues against the most relevant GNB, i.e., *E. coli*. First, we screened the efficacy of α-MSH and its four cationic analogues and studied their bactericidal kinetics against both exponential and stationary phase cells of *E. coli*. Next, we performed several fluorescence-based assays, such as lipopolysaccharide (LPS) binding, membrane depolarization, and permeabilization, to elucidate the mechanism of action of α-MSH-based cationic peptides in *E. coli* cells.

## Results

### Chemistry of the α-MSH analogues used in this study

The sequences of the peptides used in this study are shown in Table 1. Lysine (K) and Alanine (A) amino acids were used systematically to enhance the cationic charge of the peptides by replacing the uncharged polar amino acid Serine (S) at positions 1 and 3 and anionic Glutamic acid (E) at position 5 of the parent peptide α-MSH^20,21^.

**Table 1:** Name, sequence and charge of α-MSH and its derivative analogues.

Peptides	Sequence	Charge	References
α-MSH	Ac-S^1^-Y^2^-S^3^-M^4^-E^5^-H^6^-F^7^-R^8^-W^9^-G^10^-K^11^-P^12^-V^13^-NH_2_	+ 1	^21,45^
K-MSH	Ac-**K**^1^-Y^2^-S^3^-M^4^-E^5^-H^6^-F^7^-R^8^-W^9^-G^10^-K^11^-P^12^-V^13^-NH_2_	+ 2	^20^
KK-MSH	Ac-**K**^1^-Y^2^-**K**^3^-M^4^-E^5^-H^6^-F^7^-R^8^-W^9^-G^10^-K^11^-P^12^-V^13^-NH_2_	+ 3	^20^
KKA-MSH	Ac-**K**^1^-Y^2^-**K**^3^-M^4^-**A**^5^-H^6^-F^7^-R^8^-W^9^-G^10^-K^11^-P^12^-V^13^-NH_2_	+ 4	^20^
KKK-MSH	Ac-**K**^1^-Y^2^-**K**^3^-M^4^-**K**^5^-H^6^-F^7^-R^8^-W^9^-G^10^-K^11^-P^12^-V^13^-NH_2_	+ 5	^20^

The bold residues indicate the alterations in different analogues from the native peptide.

### Killing kinetics of α-MSH-based peptides against the exponential phase *E. coli* cells

**Figure 1:**
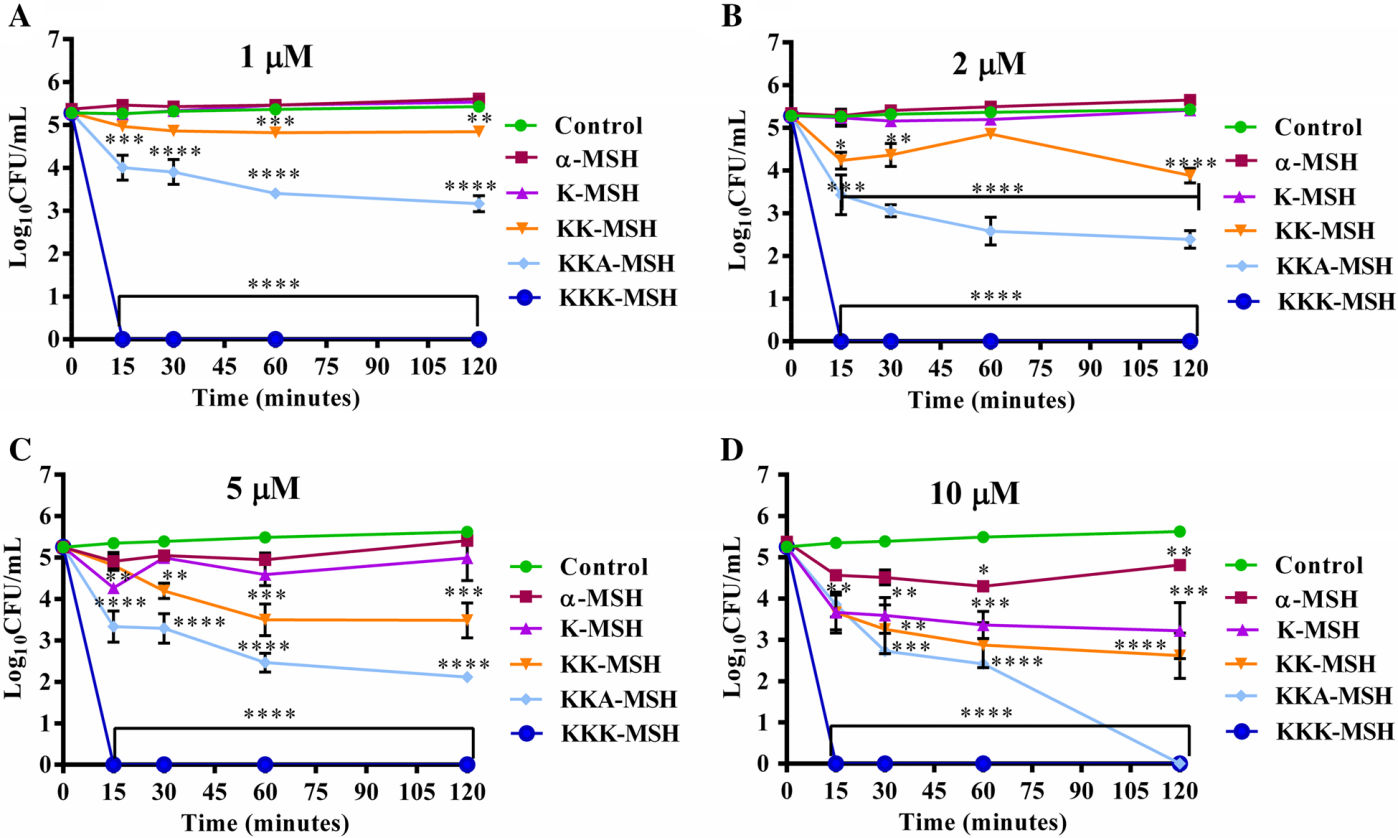
Killing kinetics of α-MSH and its cationic analogues at different concentrations, (**A**) 1 µM, (**B**) 2 µM, (**C**) 5 µM, and (**D**) 10 µM, of peptides against logarithmic phase *E. coli* ATCC 25922. The graphs were generated from the representative values (average ± SE) of three independent experiments (*P < 0.05, **P < 0.01, ****P < 0.0001 compared to untreated control).

The antimicrobial activity of α-MSH and its four analogues with gradually increasing cationicity (Table 1), namely, K-MSH, KK-MSH, KKA-MSH, and KKK-MSH, was determined for 2 h at concentrations ranging from 1 to 10 µM against the exponentially grown *E. coli* cells. An inverse relationship was observed between the decrease in cell viability and the cationic charge of the peptides. For example, while at low concentrations (1 µM and 2 µM), α-MSH did not show any activity, and its analogues with lower cationicity, i.e., K-MSH (+ 2) and KK-MSH (+ 3), showed a very slight decrease in the cell viability (Fig. 1A,B), the higher cationic charge analogues showed efficient killing efficacy. At 1 and 2 µM concentrations, KKA-MSH (+ 4) showed 1.8 ± 0.1 and 2.7 ± 0.3 log reductions, respectively, in cell viability upon incubation for 1 h, which was further reduced to 2.1 ± 0.1 and 2.9 ± 0.2 logs at 2 h incubation. Most importantly, the analogue with the highest cationic charge, KKK-MSH (+ 5), showed complete eradication of the cells within 15 min of incubation at the concentrations of 1 µM and 2 µM (Fig. 1A,B).

A relative increase in the killing by α-MSH and its analogues with lower cationicity was observed when used at higher concentrations (5 µM and 10 µM). At 5 µM, α-MSH and K-MSH showed a marginal increase in activity, whereas KK-MSH showed 1.7 ± 0.4 log reduction in cell viability after 2 h. However, KKA-MSH showed no change in its activity at 5 µM concentration (Fig. 1C). At the highest concentration tested, i.e., 10 µM concentration, even α-MSH and K-MSH exhibited 1.0 ± 0.1 and 1.9 ± 0.3 log reductions in cell viability, respectively, after 1 h of incubation. An increase in activity was also observed for KK-MSH, which caused 2.6 ± 0.5 log reductions in cell viability after 2 h of incubation (Fig. 1D). Of note, even KKA-MSH, like the other higher cationic charge analogue KKK-MSH, completely removed the cells after 2 h of incubation at the concentration of 10 µM.

Thus, among the analogues, KKK-MSH was the most effective peptide, which achieved the bactericidal effect (> 3 log reduction) by killing all 10^5^ CFUs within 15 min, even at 1 µM concentration. The second most efficacious peptide was KKA-MSH, which also accomplished bactericidal effect at 2, 5, and 10 µM concentrations upon 1 h of incubation.

### Effect of cell density on killing efficacy of the most potent analogues: KKA-MSH and KKK-MSH

**Figure 2:**
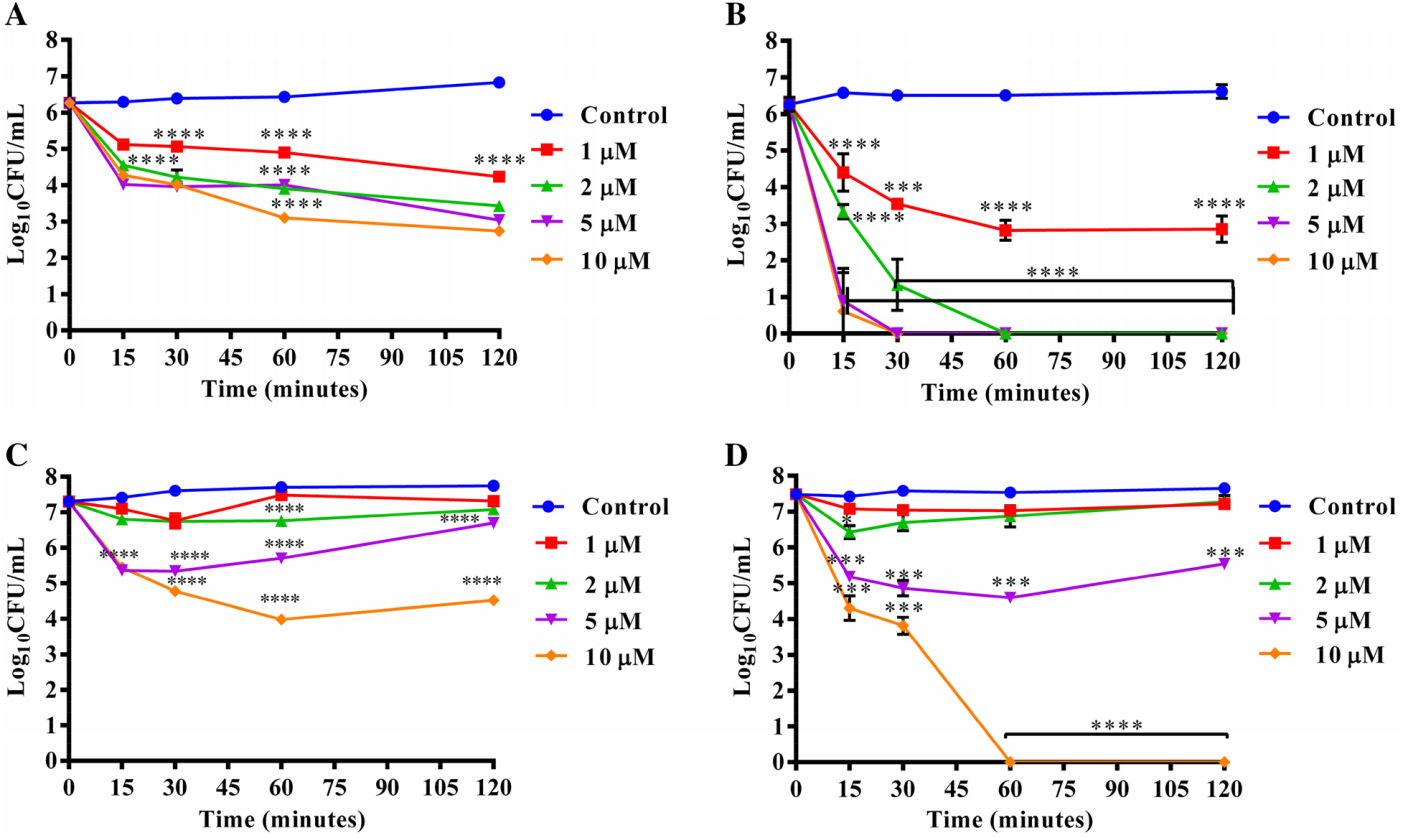
Effect of higher bacterial cell densities of logarithmic phase *E. coli* on the killing kinetics of (**A**) KKA-MSH and (**B**) KKK-MSH against 10^6^ CFU/mL, and (**C**) KKA-MSH and (**D**) KKK-MSH against 10^7^ CFU/mL at different concentrations of 1 µM, 2 µM, 5 µM, and 10 µM. Each data point denotes the representative values (average ± SE) from the experiment performed on three independent days ****P < 0.0001, compared to untreated control.

Inoculum-dependent antimicrobial efficacy was previously reported elsewhere where antibiotics, specifically β-lactams, show reduced effectiveness or loss of activity upon increasing the bacterial cell density^22^. Therefore, we tested the efficacy of the two most potent analogues, KKA-MSH and KKK-MSH, against *E. coli* cells at higher densities such as 10^6^ CFU/mL and 10^7^ CFU/mL, and observed a slight reduction in their potency. At 10^6^ and 10^7^ CFU/mL, KKA-MSH showed 3.5 ± 0.2 and 2.7 ± 0.02 log reduction, respectively, in cell viability at 10 µM concentration after 2 h of incubation (Fig. 2A,C). However, the most potent analogue, KKK-MSH, could still completely eradicate 10^6^ CFU/mL bacterial cells (Fig. 2B) at 2, 5, and 10 µM concentrations upon incubation for 1 h, 30 min, and 15 min, respectively, but not at 1 µM concentration. When incubated with 10^7^ CFU/mL *E. coli* cells, KKK-MSH resulted in no viable cells at 10 µM after 1 h incubation (Fig. 2D).

Overall, even at high cell density, KKK-MSH maintained its bactericidal effect at as low as 2 µM concentration.

### Effect of growth phase on the sensitivity of *E. coli* cells to cationic α-MSH analogues

**Figure 3:**
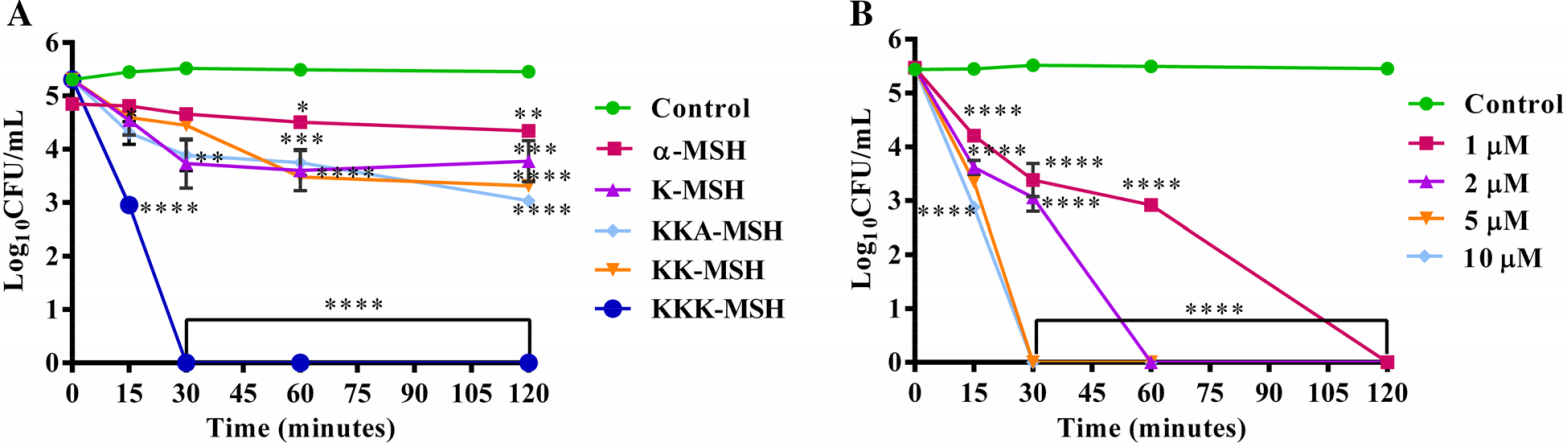
Effect of the stationary phase of *E. coli* cells on the killing kinetics of (**A**) α-MSH and its analogues at 10 µM concentration and (**B**) KKK-MSH at various concentrations. Experiment was repeated on three independent days, and the data was presented as mean ± SE. (****P < 0.0001, compared to untreated control).

As stationary phase bacterial cells are often resistant to several antibacterial agents^23^, we evaluated whether our α-MSH analogues retained their efficacy against *E. coli* cells at this particular phase in their growth. All the analogues at 10 µM concentrations were applied to the 10^5^ CFU/mL stationary phase cells of *E. coli*, and the killing activity was observed. As seen from Fig. 3A, while α-MSH showed the least killing activity, K-MSH and KK-MSH caused only 1.7 ± 0.3 and 1.8 ± 0.05 log reductions, respectively, in cell viability after 1 h of incubation. KKA-MSH, the second most cationic analogue, showed 2.2 ± 0.2 log reduction in cell viability after incubation for 2 h, which was less than its activity against exponential phase cells (> 3 log reduction in cell viability, Fig. 1D). Also, it could not obliterate the stationary phase cells after 2 h of incubation even at 10 µM concentration (Fig. 3A). On the other hand, the most cationic peptide, KKK-MSH was observed to eradicate the stationary phase cells at 10 µM concentration on incubation for 30 min (Fig. 3A). Lower concentrations of KKK-MSH resulted in an antimicrobial effect that increased with an increase in incubation time (Fig. 3B). Even at 1 µM concentration, KKK-MSH caused 2.5 log reduction in the viability of the stationary phase cells after an incubation of 1 h, followed by complete removal of the cells after 2 h. Additionally, complete killing was achieved in lesser time upon increasing the peptide concentration (Fig. 3B). Therefore, it can be inferred from the study that the most cationic analogue, KKK-MSH, is equally effective against both the stationary and exponential phases of *E. coli* cells.

### Binding of α-MSH and its analogues to *E. coli* LPS

**Figure 4:**
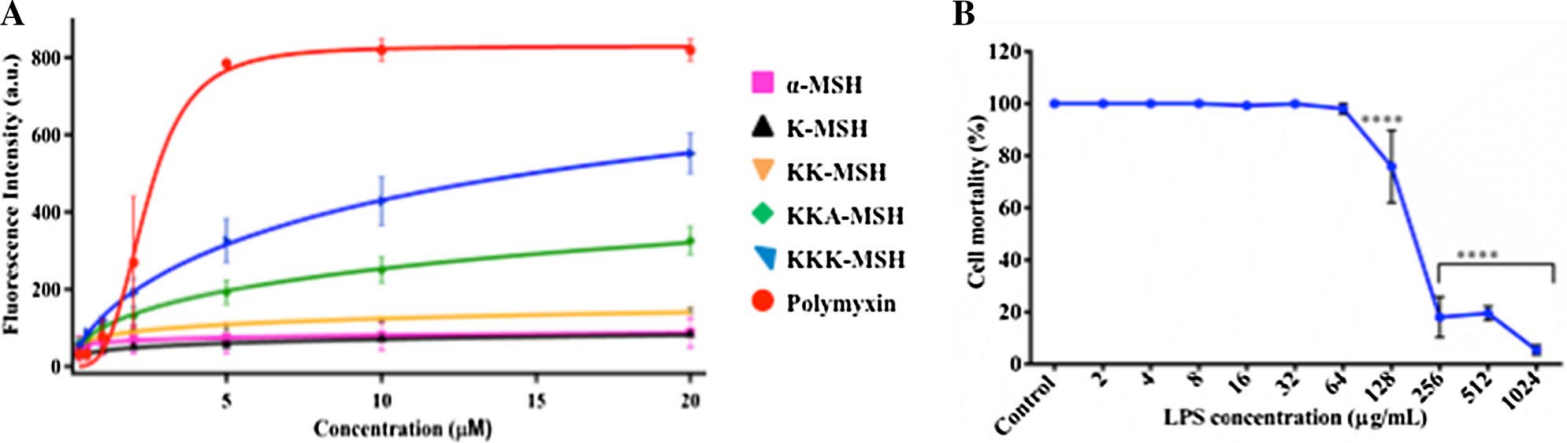
(**A**) LPS binding affinity of α-MSH and its cationic analogues was determined by BC displacement assay. The experiment was conducted on two independent days, and each data point represents the average value ± SE. The curves were fitted as described in the Materials and Methods section. (**B**) *E. coli* cell mortality at a fixed concentration (10 µM) of KKK-MSH, as a function of various lipid concentrations. The experiment was conducted on three independent days, and each data point represents the average value ± SE. The experiment was performed twice, and similar data was obtained. Representative data from one set is shown here. (**P < 0.01 and ****P < 0.0001, compared to the parent peptide at corresponding concentration).

LPS is the main component of the GNB outer membrane and imparts a negative charge to the GNB membrane; it could be a potential target for the membrane-acting cationic antimicrobial peptides like α-MSH. Therefore, we measured the binding of α-MSH and its cationic analogues to LPS (50 μg/mL from *E. coli* O55:B5) using a fluorescent dye BODIPY-TR-cadaverine (BC), which binds with the lipid A part of the LPS^24^. The binding of the peptides with LPS resulted in the displacement of this dye, increasing the fluorescence intensity of BC, thus estimating the extent of LPS-peptide binding. For this, α-MSH and its analogues at varying concentrations ranging from 0.25 to 20 µM were added to LPS bound with BC. The LPS binding affinities of α-MSH analogues were compared to that of polymyxin B, a well-known antimicrobial peptide that binds to LPS and frequently used as positive control^25^. As shown in Fig. 4A, there was a slight increase in fluorescence intensity of BC after getting displaced by α-MSH and its lower positively charged analogues K-MSH (+ 2) and KK-MSH (+ 3), whereas the two most cationic analogues, KKA-MSH and KKK-MSH, displayed a remarkable increase in fluorescence intensity of BC. The binding affinity of all the α-MSH-based peptides with LPS was dose- and cationicity-dependent (Fig. 4A and Table S1). As shown in Table S1, the calculated K_d_ (dissociation constant) value was maximum for parent peptide α-MSH, and it decreased on increasing the cationicity of the peptides. KKK-MSH (15 µM) showed the lowest K_d_ among all analogues indicating its higher binding affinity for LPS. The strong electrostatic interaction between positively charged KKK-MSH and negatively charged LPS could be the reason for its high binding affinity to LPS compared to that of the parent peptide and other analogues. The binding of peptides with LPS may be one of the critical steps in killing *E. coli* cells. To further investigate this relationship, the potency of KKK-MSH (at a fixed concentration of 10 µM) was determined in the presence of rising concentrations of free LPS (ranging from 2 to 1024 µg/mL) and presented in Fig. 4B. As anticipated, the activity of KKK-MSH was observed to be reduced to a great extent (less than 20% killing beyond 128 µg/mL of free LPS) with increasing concentrations of free LPS, thereby supporting the fact that free LPS competes with membrane-bound LPS for KKK-MSH, resulting in inefficient killing due to reduced availablility of KKK-MSH. The dissociation constant (K_d_) of α-MSH and its analogues with LPS are shown in Table S1.

### Outer membrane permeabilization of *E. coli* cells by α-MSH and its cationic analogues

**Figure 5:**
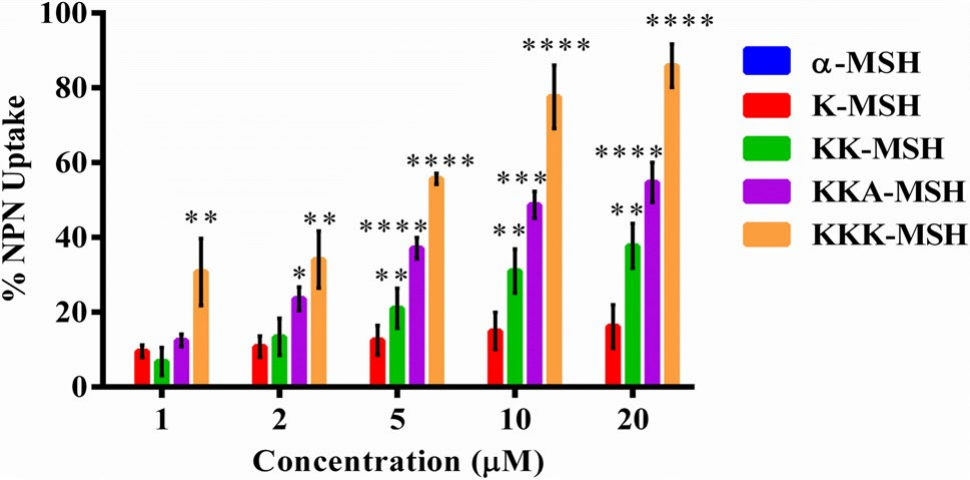
Peptide mediated outer membrane permeabilization of *E. coli*. The outer membrane permeability induced by α-MSH and its analogues was measured using the NPN (fluorescent probe) uptake assay. Data presented as average ± SE of the experiment repeated on three independent days. (**P < 0.01, ***P < 0.001, and ****P < 0.0001, compared to the parent peptide at corresponding concentration).

After confirming the binding of α-MSH and its cationic analogues with LPS in the outer membrane of *E. coli* cells, the integrity of its outer membrane upon peptide exposure was examined using a hydrophobic fluorophore, N-Phenyl-1-naphthylamine (NPN). NPN is restricted from entry into the bacterial cells due to the outer membrane, which acts as permeability barrier, and any disruption of the outer membrane by peptides would allow NPN to interact with the cell membrane’s hydrophobic components, leading to an increase in its fluorescence intensity. Therefore, the NPN uptake was measured in terms of fluorescence intensity after adding various concentrations (1 μM to 20 μM) of the peptides to *E. coli* cells pre-incubated with a fixed concentration of NPN (10 μM). The data were analyzed relative to the activity of a well-known membrane disrupting peptide, polymixin B, which was set as 100%. The membrane permeabilization was expressed in terms of %NPN uptake. An increase in NPN uptake was observed for each studied peptide with its rising concentration (Fig. 5). The analogue K-MSH showed little NPN uptake despite increasing the concentration from 1 to 20 μM with a maximum NPN uptake of 16.2%, whereas KK-MSH showed a noticeable increase in NPN uptake from 6.8% to 37.7% upon increasing its concentration. However, the second most potent analogue, KKA-MSH, showed a sharp elevation in NPN uptake from 12.5% to 55% with the increase in concentration. Notably, the most potent analogue, KKK-MSH, showed a maximum rise of 86% in NPN uptake at 20 μM concentration, almost similar to that of polymyxin B. This most cationic peptide also displayed higher NPN uptake than the other analogues at lower concentrations, i.e., 30.7%, 34%, 55.6%, and 77.6% uptake at 1, 2, 5, and 10 μM concentrations, respectively. Therefore, it can be concluded from the data that α-MSH analogues can cause disintegration of the outer membrane, and among them, KKK-MSH showed the maximum ability of the outer membrane perturbation.

### Inner membrane depolarization by α-MSH and its cationic analogues

**Figure 6:**
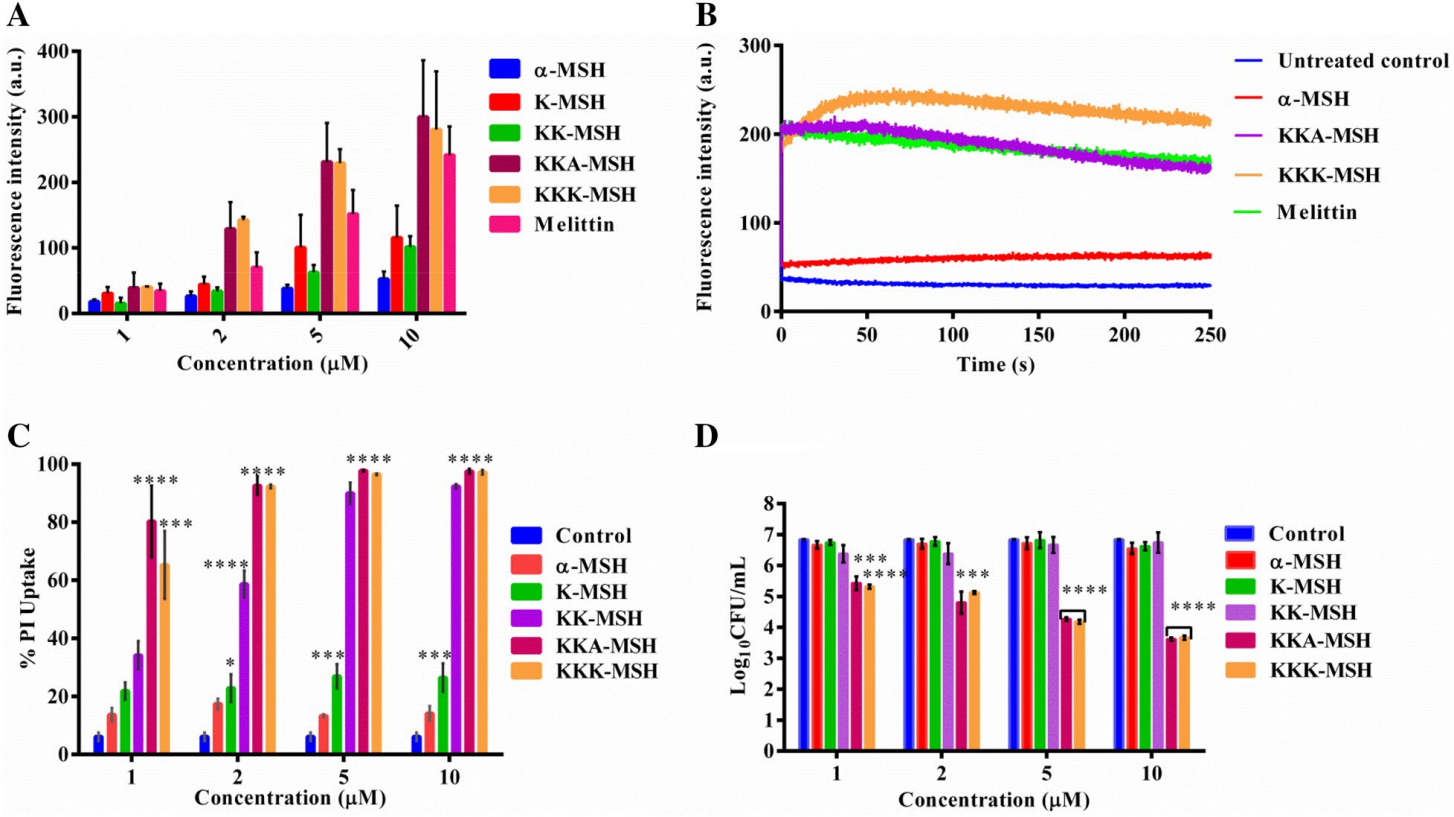
Cytoplasmic membrane disruption of *E. coli* cells. (**A**) Instantaneous depolarization (after 2 min) by α-MSH and its analogues at different concentrations using potentiometric dye DiSC_3_(5). The experiments were performed on two different days, and mean ± SE is presented here. (**B**) Depolarization kinetics of *E. coli* cells on exposure to 10 µM concentration of α-MSH, KKA-MSH, KKK-MSH, and melittin. The experiment was performed twice, and similar data was obtained. Representative data from one set is shown here. (**C**) Inner membrane permeabilization of *E. coli* cells measured by %PI uptake in cells upon treatment with α-MSH and its analogues for 1 h. (**D**) Simultaneous *E. coli* cell viability check using same cell density and peptide concentrations of PI uptake experiment. The experiment was repeated on three independent days, and the average value ± SE of each data point was presented. (**P < 0.01, ***P < 0.001, and ****P < 0.0001, compared to the value for the control at the same concentration).

It has been reported earlier that α-MSH and its cationic analogues display their efficacy by depolarizing *S. aureus* membrane^20^. Since these analogues also exerted substantial potency against *E. coli* cells and were able to bind with LPS leading to the disintegration of the outer membrane barrier, we further tested the ability of these peptides to depolarize the *E. coli* cytoplasmic or inner membrane by using potentiometric dye DiSC_3_(5). Enhancement in the relative fluorescence of the dye results from the membrane depolarization, and melittin was used as a positive control. As depicted from the results (Fig. 6A), α-MSH and its studied analogues exhibited a dose-dependent increase in fluorescence intensity of DiSC_3_(5), with the extent of depolarization being remarkable for both the higher cationic peptides KKA-MSH and KKK-MSH, compared to parent peptide α-MSH, K-MSH, and KK-MSH. Importantly, both KKA-MSH and KKK-MSH induced depolarization of *E. coli* inner membrane at a concentration as low as 2 µM; the extent of depolarization increased with the increase in peptide concentration (maximum tested peptide concentration was 10 µM).

In addition, the kinetics of membrane depolarization was monitored at 10 µM concentration of the two most cationic analogues KKA-MSH and KKK-MSH, along with α-MSH and melittin, by measuring the increase in DiSC_3_(5) fluorescence intensity for 4 min upon peptide addition. It is evident from Fig. 6B that the depolarization kinetics was rapid with a swift (within ~ 2 min) increase in fluorescence intensity upon the addition of studied peptides to the bacterial cells.

Further, we also studied the viability of dye-loaded *E. coli* cells in HEPES-glucose buffer upon peptide addition (1 µM to 10 µM) to correlate the membrane depolarization with the killing efficacy of the peptides (Fig. S1). At 5 µM, K-MSH and KK-MSH showed only 0.7 and 0.5 log reduction in cell viability, respectively, while the same concentration of KKA-MSH and KKK-MSH caused 1 and 1.73 log reduction, respectively. Upon increasing the concentration to 10 µM, K-MSH and KK-MSH resulted in 0.6 and 1.2 log reduction in cell viability, respectively, whereas KKA-MSH caused 1 log reduction and KKK-MSH showed the maximum decrease, i.e., 2.1 log reduction in cell viability after 2 min of exposure. The killing efficacy of all the analogues corresponds to their depolarization effect, confirming that the increased effectiveness of KKK-MSH is due to its enhanced cytoplasmic depolarization capacity.

### PI uptake to confirm the membrane permeabilization by α-MSH and its analogues

In order to further confirm the membrane perturbation ability of these peptides, we performed propidium iodide (PI) uptake study in *E. coli* cells upon peptide treatment. Flow cytometric analysis was done to determine %PI positive cells as shown in Fig. S2. On entering inside the cells, PI fluorescently stains DNA, indicating the disruption of cytoplasmic membrane integrity. Treatment with α-MSH and K-MSH resulted in only 13.7–14.1% and 21.8–26.5% PI positive cells, whereas incubation with KK-MSH showed a substantial increase in PI positive cells from 34.2% to 92.3% with rising peptide concentrations (Fig. 6C). Again the most cationic analogues, KKA-MSH and KKK-MSH, resulted in more than 80% PI positive cells at lower and higher concentrations (from 2 to 10 µM), supporting the results of the earlier experiments of membrane permeabilization by these analogues. Moreover, plating the cell suspension used for the PI uptake assay showed a significant reduction in the cell viability of KKA-MSH- and KKK-MSH-treated *E. coli* cells (Fig. 6D), which correlates well with this PI uptake data.

### Peptide induced morphological and intracellular changes in *E. coli* cells by SEM and TEM

**Figure 7:**
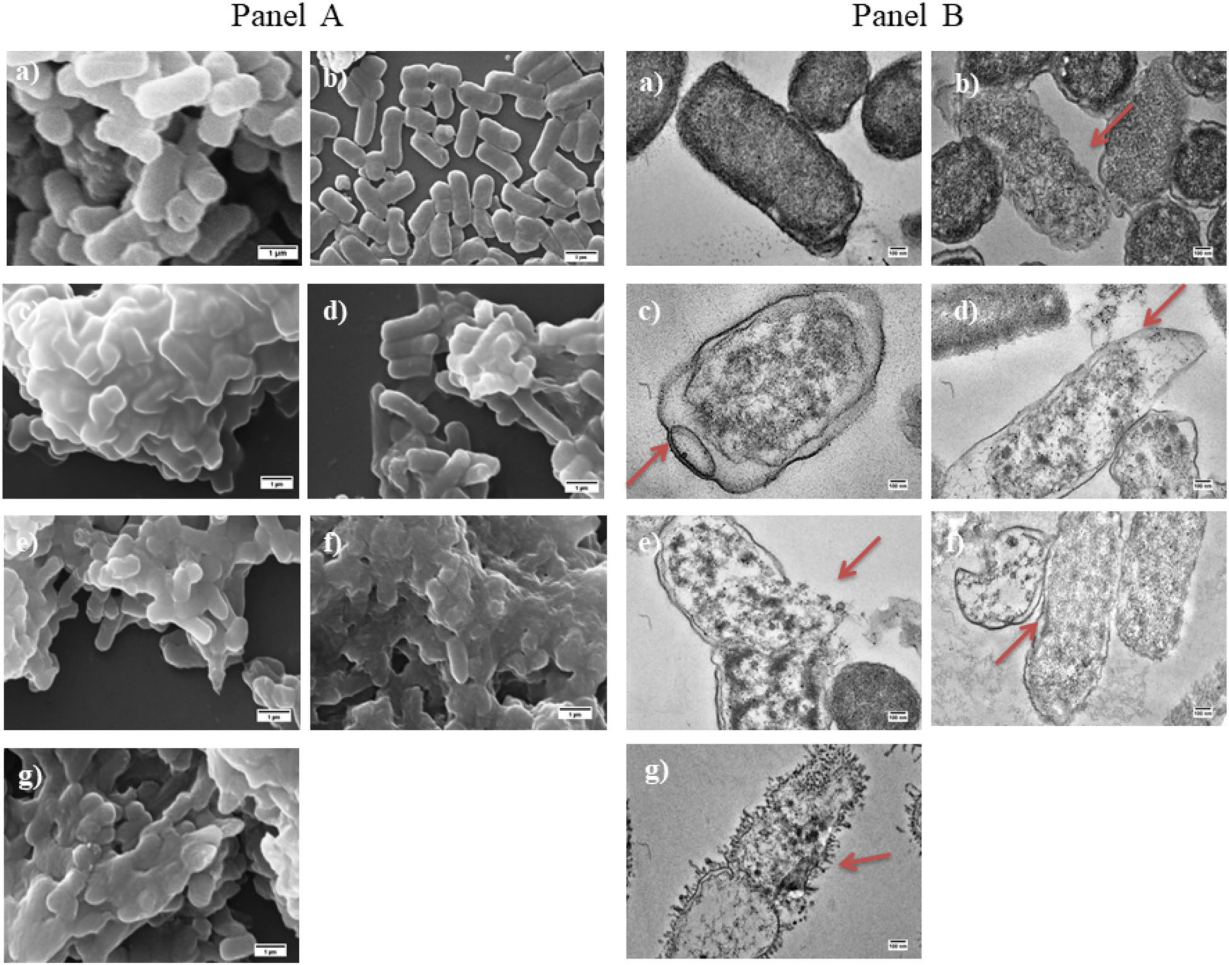
Panel (**A**): Scanning electron micrographs and Panel (**B**): Transmission electron micrographs of *E. coli* ATCC 25922 after the treatment with α-MSH and its analogues at 100 µM of concentration; (a) Untreated *E. coli* cells, and cells treated with (b) α-MSH, (c) K-MSH, (d) KK-MSH, (e) KKA-MSH, (f) KKK-MSH, and (g) 12 µg/mL of polymyxin B at a magnification of 30,000×. Scale bar = 1 µm. Red arrows indicate the major membrane alteration sites in the peptide-treated samples.

The morphology of the cells treated with α-MSH and its analogues were visualized by scanning electron microscopy (SEM). As depicted in Fig. 7(Panel A), the untreated *E. coli* cells showed a bright and smooth surface. However, after treatment with α-MSH and its analogues at 100 µM concentration for 2 h, massive distortions in the surface integrity were observed, including fragmentation of the cells and leaking out of cellular content. Similarly, the transmission electron microscopy (TEMs) images (Fig. 7, Panel B) of the *E. coli* cells incubated with the peptides showed that all the peptides caused significant membrane disruption after 2 h of exposure, wherein KKA-MSH and KKK-MSH induced the complete collapse of the *E. coli* membrane and oozing of the intracellular material.

## Discussion

The lack of effective antimicrobial agents to combat MDR Gram-negative infections is the main focus of current research worldwide. Compared to the other Gram-negative pathogens, *E. coli* infection is difficult to treat due to its diverse resistance profile against available antibiotics^7^. To seek an answer for the growing AMR problem, in our previous findings, we established a strong killing efficacy of α-MSH-based small cationic antimicrobial peptides (CAMPs) against MRSA via bacterial membrane pore formation. The primary action of a CAMP is governed by the electrostatic interaction between the cationic peptide and the anionic cell membrane of the pathogens. Therefore, an enhancement in antimicrobial activity could be anticipated with an increase in cationic charge on a CAMP; however, this effect may achieve a critical value beyond which the activity declines^26^. Encouragingly, previously, increasing the cationic charges on α-MSH by replacing the (1) anionic amino acids with Alanine, and (2) neutral amino acids with Lysine enhanced its killing efficacy against MRSA without any accompanied mammalian cell toxicity^20^. Thus, the present study was aimed to widen the antimicrobial spectrum of these cationic α-MSH analogues by establishing their antimicrobial efficacy against the Gram-negative pathogen, *E. coli*.

The antimicrobial studies in this work demonstrated that α-MSH and its four analogues, K-MSH (+ 2), KK-MSH(+ 3), KKA-MSH(+ 4), and KKK-MSH(+ 5), with increasing positive charge displayed a remarkable potency against both exponential (Fig. 1) and stationary phases (Fig. 3) of *E. coli* cells in a dose- and cationicity-dependent manner, where the most cationic analogue KKK-MSH not only showed a rapid effect but also exhibited the highest activity at a very low concentration (complete eradication at 1 µM within 15 min). This observation is consistent with our previous findings, which showed increased staphylocidal activity of α-MSH analogues upon increasing their cationic charge, most likely driven by the increased electrostatic interaction between the positively charged peptides and the negatively charged bacterial membrane. Even in the previous study, the most cationic analogue, KKK-MSH (+ 5), exhibited maximum potency against MRSA cells compared to parent peptide α-MSH^20^. Here also, the two most cationic analogues, KKA-MSH and KKK-MSH, achieved a bactericidal effect (> 3 log reduction) against *E. coli* cells at very low concentrations (5 µM and 1 µM, respectively) (Fig. 1). Moreover, KKK-MSH retained its bactericidal effect even against a very high initial inoculum (10^7^ CFU/mL) of the *E. coli* cells (Fig. 2B,D). Thus, it is worth noticing that the cationic α-MSH-based analogues displayed greater potency against *E. coli* cells in this study as compared to that against *S. aureus* in our earlier reports. This suggests the possibility of a differential and more specific interaction of these peptides with the *E. coli* cell membrane, which may have been missing in the case of *S. aureus*. Thus, we got encouraged to explore their antibacterial mechanism against GNB cells, particularly their interaction with LPS, a major component of the GNB outer membrane that also imparts an anionic charge to it. Previously, the interaction between CAMPs and LPS has been found to play a pivotal role in the cytoplasmic membrane permeabilization and subsequent GNB killing^27^. Similarly, a tryptophan-rich 13 amino acid long CAMP, indolicidin, was found to traverse the outer membrane of GNB after binding with LPS and causing the inner membrane permeabilization via the self-promoted channel formation^28^. Some CAMP such as melittin and polymyxin B bind directly to LPS, while others such as those causing the self-promoting uptake bind with divalent cation binding site on LPS molecules, causing the displacement of divalent cations that stabilize nearby LPS molecule^29^. Therefore, this study elucidated the binding activity of α-MSH and its analogues to LPS via BC displacement experiment. Our experimental results confirmed that all the cationic analogues, including α-MSH, displayed a concentration-dependent interaction with LPS; the binding efficiency of all the peptides showed a clear cationic charge-dependent relation (Fig. 4A). Importantly, binding of the most cationic analogue KKK-MSH (+ 5) to LPS is noteworthy among all the four analogues as it exhibited maximum affinity comparable to that of polymyxin B, a peptide well-known for its binding ability to LPS specifically to lipid A^25^.

This LPS-peptide binding was also confirmed by the reduction in the peptide-induced bacterial cell mortality in the presence of free LPS (Fig. 4B), presumably due to the binding competition between free and cell-bound LPS for cationic peptide, which might have effectively decreased the available concentration of the peptide. Importantly, at a high concentration of LPS (beyond 128 µg/mL), the bactericidal activity of KKK-MSH was almost completely inhibited. This is consistent with the findings of other groups where bactericidal efficacy of temporin L and a synthetic peptide 16 was inhibited in a dose-dependent manner and completely abolished by LPS at higher concentrations^30,31^.

After confirming the LPS-peptide interaction, the permeabilization of the outer membrane was established with the help of the NPN uptake assay (Fig. 5). Subsequently, the inner membrane integrity was evaluated in terms of both membrane depolarization and permeabilization induced by the peptide. Importantly, KKK-MSH was remarkable among all the analogues and displayed a rapid and strong inner membrane depolarization (Fig. 6A,B) and permeabilization (Fig. 6C,D) higher than that of melittin. Based on these results, it can be inferred that the favorable interaction of *E. coli* LPS and KKK-MSH resulted in the efficient insertion of the peptide into the cell, followed by inner membrane disruption, leading to bacterial cell death. Although similar findings were reported, showing a dose-dependent depolarizing effect of LL-37 on *P. aeruginosa* and PMAP-36, melittin, and GI24 on *E. coli* membranes^32,33^, one could still argue about how the peptide reaches the inner membrane if it gets neutralized after binding with LPS molecules associated with the outer membrane. Our study confirmed both efficient binding between studied peptides and LPS (though binding affinity of cationic amphipathic peptides may vary for free soluble LPS and cell-bound LPS depending upon the peptide-LPS ratio) as well as a rapid and strong depolarization of *E. coli* inner membrane on peptide exposure. Thus, it may be likely that the amphipathic characteristics of these peptides^20^ enabled their binding to LPS in the outer membrane as well as the transient interactions with the inner membrane, leading to bilayer disintegration by cell disruption resulting in osmotic imbalance^34,35^. Furthermore, the absence of precise pore formation, similar to gramicidin, as seen in the SEM and TEM images (Fig. 7A,B), confirmed that the α-MSH-based peptides induced cell lysis and leakage of intracellular material. Additional study is required to understand the detailed interaction of these peptides with membrane components using solid-state NMR spectroscopy^34–36^.

Collectively, it can be concluded that α-MSH and its cationic analogues exhibited promising antibacterial effects against *E. coli*, a representative GNB. They selectively targeted the LPS, a significant component of the *E. coli* outer membrane, and ultimately caused the cytoplasmic membrane disruption, resulting in cell death. Cationicity of these peptides was a key player in governing their antimicrobial effect against *E. coli* via binding with the negatively charged LPS molecules, and as such, KKK-MSH, being the most cationic analogue showed maximum potency. However, CAMPs may follow multi-target mechanisms to achieve an efficient killing; a few CAMPs have been putatively associated with DNA, RNA, and protein synthesis inhibition in GNB after crossing the membrane barriers^37^. Although targeting cell wall synthesis, DNA and protein synthesis are the supportive processes in achieving a bactericidal effect, the mechanism of cell membrane disruption is more relevant in this study, as KKK-MSH achieved complete eradication of the *E. coli* inoculum, which was well corresponding to its LPS binding ability followed by cell membrane disruption.

## Conclusion

In this study, we established the antimicrobial activity of α-MSH and its cationic analogues against Gram-negative pathogen *E. coli*. Our results suggested that the cationicity of the peptides played an essential role in their mode of action by specifically enabling their interaction with the LPS molecules abundant in the *E. coli* outer membrane. The experiments like killing kinetics, fluorescence, and microscopy studies clearly showed that α-MSH and its cationic analogues are membrane lytic peptides against both *E. coli* and *S. aureus* bacteria; however, LPS binding of these peptides was unique to *E. coli*. The most cationic analogue KKK-MSH showed the strongest affinity to LPS, leading to the rapid depolarization of the inner membrane, destruction of the inner membrane integrity, oozing out of intracellular material, followed by eventual bacterial cell death. Based on our present and previous findings, KKK-MSH can be proposed as a lead analogue of α-MSH for developing a broad-spectrum membrane-active drug with significant efficacy against clinically relevant Gram-positive and Gram-negative pathogens.

## Materials and methods

### Materials

N-phenyl naphthylamine (NPN), 3,3′-Dipropylthiadicarbocyanine iodide (DiSC_3_(5)), dimethylsulfoxide (DMSO), propidium iodide (PI), and lipopolysaccharides (LPS) were acquired from Sigma-Aldrich, India. BODIPY-TR-cadaverine (5-(((4-(4,4-Difluoro-5-(2-Thienyl)-4-Bora-3a,4a-Diaza-s-Indacene-3-yl)phenoxy)acetyl)amino)pentylamine, hydrochloride) was purchased from Thermo Fisher Scientific (India). Disodium hydrogen orthophosphate dihydrate (Na_2_HPO_4_·2H_2_O), sodium dihydrogen orthophosphate dihydrate (NaH_2_PO_4_·2H_2_O), and sodium chloride (NaCl) were purchased from Qualigens Fine Chemicals, India. 4-(2-hydroxyethyl) piperazine-1-ethanesulfonic acid (HEPES) was purchased from SRL (India). Luria Bertani (LB) was procured from Difco (India), and agar powder was purchased from Himedia Laboratories, India.

### Peptides

α-MSH, polymyxin B, and melittin were purchased from Sigma-Aldrich, India. The designed peptide analogues (HPLC purity > 98%) were custom synthesized by Biochain Incorporated, India. The concentration of the peptides was determined by measuring the UV absorbance (through UV–Visible absorption spectrophotometer, Shimadzu) of Trp and Tyr amino acids at 280 nm, using the theoretical extinction coefficient (ε) 6.65 × 10^3^ M^−1^ cm^−1^ for both Trp and Tyr.

### Bacterial strain

For this study, a well-characterized strain of *E. coli* (ATCC 25922) was used. The strain was stored at − 80 °C in 15% (v/v) glycerol until sub-cultured onto LB agar plates for use.

## Methodology

### Killing assay

The antibacterial activity of α-MSH and its cationic analogues was determined against both logarithmic (2–3 h) and stationary (16–18 h) phase of *E. coli* cells in LB medium^16^. The bacterial suspension was pelleted, washed, and diluted in HEPES-glucose buffer (5 mM HEPES, 20 mM glucose, pH 7.4) to adjust the cell density by measuring the OD = 0.5 (10^8^ CFU/mL) at 600 nm using a spectrophotometer. This suspension was used to obtain the desired inoculum sizes (10^5^ CFU/mL, 10^6^ CFU/mL, and 10^7^ CFU/mL). The inoculums were exposed to various concentrations of peptides (1 µM, 2 µM, 5 µM, and 10 µM) and incubated at 37 °C. Untreated *E. coli* cells were also included as a control. At selected time points (0 min, 15 min, 30 min, 60 min, and 120 min), aliquots were taken out, and survival of *E. coli* cells was determined by quantitative plating on LB agar after appropriate dilution in the same buffer. The plates were incubated overnight at 37 °C, followed by colony counting. Each experiment was performed at least three times on three independent days.

As described in our earlier reports^17,20,38^, the minimum inhibitory concentration (MIC) of α-MSH and its cationic analogues could not be determined as their antimicrobial activity got mitigated in the presence of the growth medium used in the broth microdilution assay.

### Outer membrane permeabilization assay

The outer membrane permeabilization assay was done as described in the literature with slight modifications^39^. Briefly, bacterial cells were grown to mid-log phase in LB medium, washed and diluted to 10^8^ CFU/mL in HEPES-glucose buffer. Next, this bacterial suspension was treated with 10 μM NPN probe and subsequently challenged with increasing concentrations of peptides (1 µM to 20 µM). Changes in the fluorescence intensity of NPN were measured with the help of Shimadzu RF-5301 PC spectrofluorimeter (excitation at 350 nm and emission at 420 nm). Increased intensity of emission resulted from the damage to the bacterial outer membrane and was measured until no further increase in fluorescence was observed. NPN uptake was calculated using the following formula:

$$\% \;{\text{NPN}}\;{\text{uptake}} = \left( {{\text{F}}_{{{\text{obs}}}} - {\text{F}}_{0} } \right)/\left( {{\text{F}}_{100} - {\text{F}}_{0} } \right) \times 100$$

where F_obs_ is the NPN fluorescence intensity after the peptide treatment, F_0_ is the NPN fluorescence with untreated *E. coli* cells, and F_100_ is the NPN fluorescence in the presence of 10 µM polymyxin B (a positive control).

### Membrane depolarization assay

The membrane depolarization of *E. coli* cells was determined using a potentiometric fluorescent probe DiSC_3_(5), as described elsewhere^40^. Briefly, mid-log phase *E. coli* cells were diluted to 10^6^ CFU/mL in the same HEPES-glucose buffer and were incubated with DiSC_3_(5) and EDTA at 0.4 µM and 0.2 mM concentrations, respectively, for 1 h in the dark at 37 °C, till the maximum uptake of the probe occurred. Increasing concentrations of the studied peptides from 1 µM to 10 µM were then added to these dye-loaded cells in a quartz cuvette, and the fluorescence intensity was measured on spectrofluorimeter (Shimadzu RF-5301 PC) set as excitation at 622 nm and emission at 670 nm. The increase in fluorescence intensity accounted for the dissipation of membrane potential caused due to peptide treatment. Cells treated with 10 µM melittin were used as a positive control. Simultaneously, the dye-loaded cells were plated onto LB agar to estimate bacterial cell death.

### Membrane permeabilization assay

Membrane permeabilization of *E. coli* cells was determined via propidium iodide (PI) uptake assay through flow cytometry as described previously with slight modifications^41^. Briefly, mid-log phase *E. coli* cells were adjusted to 10^6^ CFU/mL in the HEPES-glucose buffer. Next, cells were incubated with propidium iodide (PI) at 1.3 µg/mL concentration for 30 min in the dark. After incubation, PI-loaded cells were treated with a range of peptide concentrations, i.e., 1 µM, 2 µM, 5 µM, and 10 µM, at 37 °C for 1 h, and PI fluorescence intensity was measured through Becton Dickinson (BD) FACS verse (San Jose, CA) flow cytometer using excitation at 544 nm and emission at 620 nm. The *E. coli* cells that showed fluorescence intensity more than 10 units (arbitrary) were considered stained with PI.

### LPS binding assay

The binding of α-MSH and its analogues to LPS was checked using BODIPY-TR-cadaverine (BC) as a fluorescent probe^24,42^. BC fluorescence decreases when bound to cell-free LPS and increases when it is freely accessible in solution. LPS (50 μg/mL) from *E. coli* O55:B5 (Sigma-Aldrich, India) was incubated with 5 µM of BC in 50 mM Tris buffer (pH 7.4) for 4 h. Next, 800 µL of this LPS-BC mixture was added to varying concentrations (0.25 µM to 20 µM) of the peptides in a quartz cuvette, and the BC fluorescence was measured on a spectrofluorimeter (Shimadzu RF-5301 PC) with excitation at 580 nm and emission at 620 nm. Each experiment was done in duplicate independently. Increase in fluorescence intensities with increase in concentration of peptides were plotted and the dissociation constant (K_d_) was calculated from each individual data set and is presented as mean value ± SD using Igor Pro 6.36 (Y = Bmax × Xˆh/(K_d_ˆh + Xˆh); where X: concentration of peptide (µM), Y: fluorescence intensity (a.u.), h: Hill slope)^43^. The molecular mass of LPS was taken as 20,000 Da as described elsewhere, and its concentration was calculated accordingly^44^.

### LPS competitive inhibition assay

To determine whether LPS could influence the peptide’s antimicrobial activity, the potency of the most charged analogue, KKK-MSH, was evaluated against *E. coli* in the presence of LPS, using the protocol reported elsewhere^30^. Peptide and LPS from *E. coli* O55:B4 (Sigma-Aldrich, India) in equal volume (50 µL) were incubated in a 96-well sterile plate at 37 °C for 1 h. After that, 100 μL of mid-log *E. coli* cells were added (at a final concentration of 10^5^ CFU/mL) to the LPS-peptide mixture for 90 min at 37 °C. The peptide concentration was kept constant at 10 µM, and the concentration of LPS was varied from 2 to 1024 μg/mL. After incubation, 50 μL of the reaction mixture from each well was diluted with an appropriate amount of HEPES-glucose buffer, spread on LB agar plates, and kept at 37 °C for 16 h. Then on the next day, colonies were counted and compared with control (in the absence of LPS, cells treated with only peptide were considered 100% mortal). Mortality was calculated using the following formula:

$$\begin{aligned} & \% \;{\text{survival}} = \left[ {\left( {{\text{CFU/mL}}\;{\text{in}}\;{\text{treated}}\;{\text{sample}}} \right)/\left( {{\text{CFU/mL}}\;{\text{in}}\;{\text{untreated}}\;{\text{sample}}} \right)} \right] \times 100 \\ & {\text{Cell}}\;{\text{mortality}}\;{(\% )} = 100 -\% \;{\text{survival}} \\ \end{aligned}$$

This experiment was performed on three independent days.

### Morphological changes study via

#### Scanning electron microscopy (SEM)

Changes in the morphology in *E. coli* due to the treatment with peptides were studied by SEM using the procedure described earlier^16^. *E. coli* cells were grown to logarithmic phase in LB medium, washed thrice, resuspended in HEPES-glucose buffer, adjusted to 10^8^ CFU/mL, and treated with each test peptide independently at 100 µM concentration for 2 h. The peptide-treated cells were washed with PB buffer (10 mM Sodium-phosphate buffer, pH 7.4) thrice to remove any salt and were further fixed with 2.5% glutaraldehyde in 0.1 M PB buffer overnight at 4 °C. After fixation, cells were washed with the same buffer twice. Next, the cells were dehydrated and finally dried in a desiccator under vacuum. The samples were viewed via SEM (Carl Zeiss EVO40) after coating with 20 nm gold particles using an automatic sputter coater (polaron OM-SC7640).

#### Transmission electron microscopy (TEM)

To detect ultra-structural changes in *E. coli* on peptide treatment, TEM was used^16^. In brief, the mid-log *E. coli* cells (10^8^ CFU/mL) were exposed with 100 µM concentration of all peptides independently for 2 h at 37 °C. After treatment, the cells were washed with PB buffer and fixed with 2.5% glutaraldehyde overnight at 4 °C. Next, the samples were post-fixed in 1.0% OsO_4_ and stained with uranyl acetate. After fixation, cells were dehydrated in a series of graded acetone and embedded in epoxy resin. Thin sections were made using a microtome (Leica EM UC6), which were transferred onto copper grids and stained with uranyl acetate followed by lead citrate. Samples were viewed via a transmission electron microscope (JEOL 2100F) to detect the ultrastructural and other morphological changes in the bacterial cells.

### Statistical analysis

Statistical analysis was carried out using one-way ANOVA to determine the significance of the experiment among the groups. The data were analyzed using Graph Pad Prism 6. Quantitative data were expressed as mean ± standard error of mean (SE), and *P* < 0.05 was considered statistically significant. The average value obtained for the peptide-treated samples was significantly different from that of the control at the equivalent time points (*P* < 0.05), thus the null hypothesis was rejected.

## Supplementary Information

Supplementary Information.
