# Supplementary material for: Binding of cationic analogues of α-MSH to lipopolysaccharide and disruption of the cytoplasmic membranes caused bactericidal action against Escherichia coli
Source: Sci Rep. 2022 Feb 7;12:1987. doi: 10.1038/s41598-022-05684-z (PMC8821551; doi:10.1038/s41598-022-05684-z)
Supplement: Supplementary file 1 — Supplementary Information. [file 41598_2022_5684_MOESM1_ESM.docx]

**ELECTRONIC SUPPLEMENTARY INFORMATION (ESI)**

**Binding of cationic analogues of α-MSH to lipopolysaccharide and disruption of the cytoplasmic membranes caused bactericidal action against**

***Escherichia coli***

Kanchan Tiwari, Madhuri Singh*, Prince Kumar & Kasturi Mukhopadhyay*

Antimicrobial Research Laboratory, School of Environmental Sciences, Jawaharlal Nehru University, New Delhi.

*Correspondence: [chauhan.madhuri@gmail.com](mailto:chauhan.madhuri@gmail.com) and [kasturim@mail.jnu.ac.in](mailto:kasturim@mail.jnu.ac.in)

**Table of contents**

1. **Figure S1:** Survival of *E. coli* cells corresponding to cytoplasmic depolarization
2. **Figure S2:** Histogram of *E. coli* cells treated with α-MSH and its analogues.
3. **Table S1:** The dissociation constant (K_d_) of α-MSH and its analogues with LPS.

**

**

**Figure S1**: **Survival of dye-loaded cells (10^6^) treated with various concentrations of peptides that correspond to the inner membrane depolarization.** The dye-loaded *E. coli* cells suspended in HEPES-glucose buffer (pH 7.2) were incubated with different concentration of α-MSH and its analogues for 2 minutes and further diluted in the same buffer and plated on LB agar plates. The plates were further incubated at 37ºC for 16 h. Next day the colonies were counted.^1^ The experiment was performed on two different days and the representative values (mean ± SE) are shown in graph. (⁎, P < 0.05; ⁎⁎, P < 0.01, compared to that of control at the same concentration).


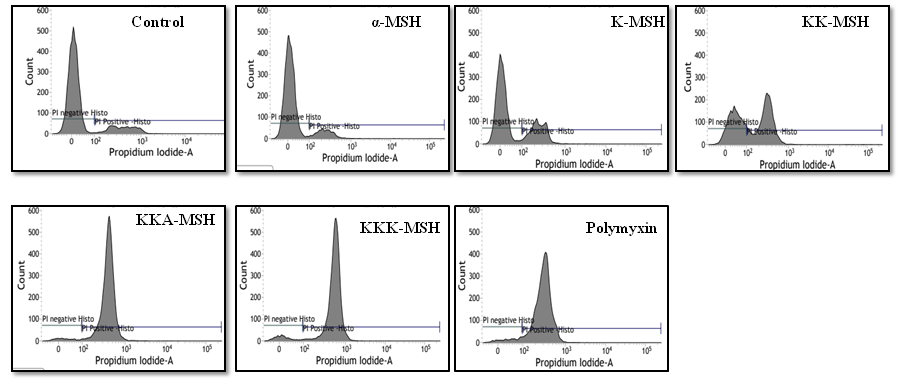


**Figure S2: Histograms of *E. coli* cells treated with 2 µM concentration of α-MSH and its analogues and 1 µM concentration of polymyxin B for 1 h.** Towards monitoring the membrane permeabilizations of *E. coli* cells, propidium iodide (PI) uptake assay through flow cytometry was performed using the previously standardized method with mild modifications ^2^. Briefly, mid-log phase grown *E. coli* cells (10^6^ CFU/mL) suspended in the HEPES-glucose buffer were incubated with propidium iodide (PI) at a concentration of 1.3 µg/mL for 30 min in dark. After incubation, PI loaded cells were treated with a range of peptide concentrations; 1 µM, 2 µM, 5 µM and 10 µM at 37 °C for 1 h, and PI fluorescence intensity was measured through Becton Dickinson (BD) FACS verse (San Jose, CA) flow cytometer using excitation at 544 nm and emission at 620 nm. The *E. coli* cells which showed fluorescence intensity more than 10 units (arbitrary) were considered as stained with PI.

**Table S1: The dissociation constant (K_d_) of α-MSH and its analogues with LPS.** K_d_ was determined by fitting the data of each individual experiments shown in Fig 4A with the hill equation (Y = Bmax × Xˆh/(K_d_ˆh + Xˆh); where X: concentration of peptide (µM), Y: fluorescence intensity (a. u.), h: Hill slope)^3^ using Igor Pro 6.36 software and presented as mean K_d_ ± SD in table S1.

| **Peptides** | **K_d_ (µM)** |
| --- | --- |
| α-MSH | 166.0 ± 41.6 |
| K-MSH | 143 ± 16.2 |
| KK-MSH | 98.7 ± 22.7 |
| KKA-MSH | 77.5 ± 16.3 |
| KKK-MSH | 9.8 ± 6.3 |
| Polymyxin | 2.4 ± 0.84 |

**References**

1. Singh, J. *et al.* Enhanced cationic charge is a key factor in promoting staphylocidal activity of α-Melanocyte stimulating hormone via selective lipid affinity. *Sci. Reports.* **6**, 31492 (2016).

2. Tyagi, P., Singh, M., Kumari, H., Kumari, A. & Mukhopadhyay, K. Bactericidal activity of curcumin I Is associated with damaging of bacterial membrane. *PLoS ONE* **10**, e0121313 (2015).

3. Yoshikawa, C., Ishida, H., Ohashi, N. and Itoh, T. Synthesis of a coumarin-based PPARγ fluorescence probe for competitive binding Assay. Int. J. Mol. Sci. **22**, 4034 (2021).
